# Effect of flunarizine on defibrillation outcomes and early refibrillation in a canine model of prolonged ventricular fibrillation

**DOI:** 10.1113/EP087068

**Published:** 2019-10-09

**Authors:** Chaofan Xing, Qi Jin, Ning Zhang, Shaohua Liu, Changjian Lin, Qiong Wu, Qingzhi Luo, Ao Liu, Liqun Wu

**Affiliations:** ^1^ Department of Cardiology Shanghai Rui Jin Hospital Shanghai Jiao Tong University School of Medicine Shanghai China

**Keywords:** defibrillation, delayed after‐depolarization, ventricular fibrillation

## Abstract

**New Findings:**

- **What is the central question of this study?**
  Can successful electrical shock in combination with a delayed after‐depolarization (DAD) blocker suppress early refibrillation episodes following long duration ventricular fibrillation (LDVF)?
- **What is the main finding and its importance?**
  Flunarizine significantly reduced the activation of LDVF and early ventricular fibrillation (VF) recurrence following LDVF, suggesting that DADs potentially contribute to refibrillation in prolonged VF. Thus, DAD inhibition can be used as an adjunctive therapy for electrical defibrillation to treat prolonged VF and suppress refibrillation following LDVF.

**Abstract:**

This study attempts to detect changes in the defibrillation threshold (DFT) at different stages of ventricular fibrillation (VF) (short duration VF, SDVF; long duration VF, LDVF) and during early refibrillation following successful defibrillation of LDVF by giving flunarizine, a blocker of delayed after‐depolarizations (DADs). Twelve beagles were divided into two groups (the control group, *n* = 6; and the flunarizine group, *n* = 6). Two 64‐electrode basket catheters were deployed into the left and the right ventricles for global endocardium mapping. The DFTs of SDVF and LDVF were determined at 20 s and 7 min, respectively, after VF induction in each group. Any refibrillation episodes were recorded within 15 min after the first successful defibrillation of LDVF. In the flunarizine group, the SDVF‐DFT values before and after the drug were not significantly different. The 7 min LDVF‐DFTs were markedly reduced by 26% (*P *< 0.05, the control group) and 38% (*P *< 0.01, the flunarizine group) compared to the 20 s SDVF‐DFTs within each group. The difference between SDVF‐DFT and LDVF‐DFT after flunarizine was larger than that in the control group (213 ± 65 *vs*. 120 ± 84 V, *P *< 0.05). The number of refibrillation episodes per animal (1.3 ± 1.0) following successful defibrillation of LDVF after flunarizine was 48% of that in controls (2.7 ± 2.0, *P *< 0.05). The effect of flunarizine on SDVF‐DFT and LDVF‐DFT indicates that the role of DADs in the defibrillation mechanism may differ as VF continues. Flunarizine significantly reduced early VF recurrence following LDVF, suggesting that DADs potentially contribute to refibrillation in a canine model of prolonged VF.

## Graphical abstract

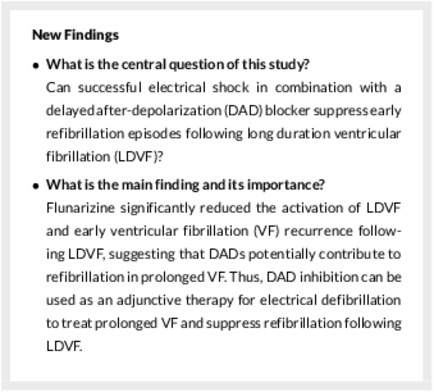

## 1 INTRODUCTION

Sudden cardiac arrest (SCA), commonly caused by ventricular fibrillation (VF), is responsible for more than 500,000 deaths every year in China (Hua et al., 2009). Many studies have been performed to investigate the defibrillation efficacy and mechanism of SDVF (VF < 1 min) because this short interval is important for an implantable cardioverter defibrillator. However, recent studies (Dosdall et al., 2010; Huang et al., 2014; Li et al., 2013; Li, Jin, Huang, Cheng, & Ideker, 2008) and our previous studies (Jin et al., 2013; Lin et al., 2014) have shown that the maintenance and termination of VF lasting more than 1 min (LDVF) differed from the maintenance and termination of SDVF. Since VF‐induced intracellular calcium overload plays an important role in defibrillation (Zaugg et al., 1998), reduction in or normalization of intracellular calcium concentration by suppressing delayed after‐depolarizations (DADs) should improve the defibrillation efficacy (Kojima, Wu, Wikman‐Coffelt, & Parmley, 1995). DADs begin during phase 4, after repolarization is complete but before another action potential would normally occur via the normal conduction systems of the heart. DADs are due to elevated cytosolic calcium concentrations, classically seen with digoxin toxicity. However, the effects of flunarizine (a DAD blocker inhibiting Ca^2+^ channels) on the SDVF defibrillation threshold (DFT) are inconsistent (Chattipakorn & Ideker, 2003; Zheng, Walcott, Smith, & Ideker, 2005). A global epicardial electrical mapping study in pigs did not find evidence that activation following failed defibrillation was caused by DADs (Zheng et al., 2005). Recently, Li *et al*. documented that DADs played a role in the maintenance of LDVF by administrating flunarizine (Li et al., 2013). Flunarizine, a selective Ca^2+^ channel antagonist, has been shown to block L‐type and T‐type Ca^2+^ channels and is thought to selectively block the accumulation of intracellular calcium during calcium overload (Holmes, Brogden, Heel, Speight, & Avery, 1984). In animal models, flunarizine has been shown to suppress DADs or DAD‐dependent ventricular tachycardias (VTs) induced either by ouabain (Rosen & Danilo, 1980; Vos, Gorgels, Leunissen, & Wellens, 1990) or by catecholamines (Donck, Pauwels, Vandeplassche, & Borgers, 1986). Additionally, flunarizine does not affect normal or abnormal automaticity, as shown in studies of sinus rhythm and early post‐myocardial infarct VT in canine models and barium‐depolarized canine Purkinje fibres (Vos et al., 1990).

However, the role of DADs in LDVF defibrillation remains unclear. Therefore, the first objective of this study was to detect changes in defibrillation efficacy at different stages of VF by administering flunarizine.

While refibrillation following SDVF is rare, especially in healthy hearts, many individuals refibrillate for at least one episode after successful defibrillation of LDVF (Allred et al., 2008; van Alem, Post, & Koster, 2003). DAD‐mediated triggered activities (TAs) secondary to spontaneous sarcoplasmic reticulum (SR) Ca^2+^ release underlie the mechanism of post‐shock arrhythmias, including VT and VF (Maruyama et al., 2010). VF recurrence is thought to be related to the persistent presence of triggers and pre‐existing substrates induced by prolonged global ischaemia (Wu, Lin, Hsieh, Chen, & Ting, 2008). The results of these previous studies indicated that electrical shock alone cannot eliminate the substrates during LDVF and that DADs may play a role in the refibrillation of LDVF. Therefore, the second objective of this study was to determine whether successful electrical shock in combination with a DAD blocker (flunarizine) could significantly suppress early refibrillation episodes following LDVF.

## 2 METHODS

All animals were purchased from Shanghai Jiao Tong University Agriculture College (China) and raised under controlled conditions in the Department of Animals for Scientific Research, Shanghai Jiao Tong University School of Medicine. All procedures in this study were approved by Shanghai Jiao Tong University School of Medicine Animal Care and Use Committee (Protocol Registry No.: A‐2015‐014) in strict accordance with the recommendations in the *Guide for the Care and Use of Laboratory Animals* of the National Institutes of Health (eighth edition, 2011). All experiments comply with the principles and regulations, as described by Grundy (2015). One animal per cage was housed in a temperature‐regulated room (22–25°C) at 30–40% relative humidity with a 12:12 h light–dark cycle and unlimited access to water and food. The experimental procedures began after the institutional veterinarian and animal care staff examined the animals and confirmed that the animals were in good health.

### 2.1 Animal preparation

Twelve beagles (12 ± 1.1 kg, mean ± SD) of both sexes were equally and randomly divided into two groups (the control group and the flunarizine group). Intramuscular injection of ketamine (10 mg kg^−1^) and atropine (0.04 mg kg^−1^) was used for anaesthesia induction. After endotracheal tube placement, anaesthesia was maintained with propofol (1–2 mg kg^−1^ min^−1^ i.v.) and inhaled isoflurane (1–3%), and fentanyl (1–2 µg kg^−1^ i.v. intermittently repeated as needed) was used to maintain analgesia. The dogs were placed in the supine position and fixed on the experimental table. The skin was prepared and electrodes placed on the skin and the leads connected. Core body temperature, arterial blood pressure (BP), arterial blood gases, cardiac electrical activity and serum electrolytes were monitored and maintained within normal ranges throughout the study. The heart was exposed through a median sternotomy and supported in a pericardial sling. At the conclusion of the experiment, the animals were killed by intravenous administration of a lethal dose of sodium pentobarbital (150 mg kg^−1^) under general anaesthesia.

### 2.2 Electrode placement

A multielectrode basket (Constellation Catheter, model US8031U, Boston Scientific, Natick, MA, USA) was introduced through the left carotid artery into the left ventricle (LV). Another basket catheter was applied through the right jugular vein into the right ventricle (RV). Each catheter contained eight splines, each with eight electrodes approximately 2 mm apart. These two catheters were used to map the ventricles simultaneously. Our previous study described the detail of how the location of the electrodes in the ventricles was determined (Jin et al., 2013). A catheter (model 80993, IBI, St Jude Medical Inc., Little Canada, MN, USA) was placed for defibrillation with the negative electrode in the RV apex and the positive electrode in the superior vena cava.

### 2.3 QT interval, ventricular effective refractory period and activation recovery interval measurements

The QT interval and the corrected QT (QTc, QT/RR^1/2^) intervals from ECGs were determined in sinus rhythm before and after flunarizine. The electrode at the tip of the basket catheter at the RV apex was used for stimulation (MicroPaceIII, EPS320 Cardiac Stimulator, Micropace EP, Inc., Santa Ana, CA, USA). Pacing stimuli were delivered at twice the diastolic threshold. The ventricular effective refractory period (VERP), which is the time following the triggering of an action potential when the cell membrane has changed to an unexcitable state and is gradually restored to the resting (excitable) state, was determined by an S1S2 stimulating programme with eight S1 stimuli of 300 ms followed by an S2 stimulus. The S1–S2 interval started from 250 ms and then decreased in 10 ms steps until S2 could not capture the ventricle. Then, the S1–S2 interval was reset to 10 ms more than this non‐capture interval and was decreased in 2 ms decrements until S2 again failed to capture the ventricles. This S1–S2 interval was defined as the VERP. The activation recovery interval (ARI), defined as the interval between the activation time and the recovery time calculated from the unipolar electrogram, is related to the VERP. The ARI was measured at the last stimuli in a series of 30 stimuli in 300 ms. The activation time on the unipolar electrograms was defined as the steepest downslope of the QRS complex, and the recovery time was defined as the fastest upslope of the T wave. The ARI was defined as the interval between the activation time and the recovery time (Jin, Chen, Smith, Ideker, & Huang, 2008).

### 2.4 VF induction and DFT measurement

After the measurement of VERP and ARI, VF was induced by a 30 Hz stimulation delivered through one of the basket electrodes in the RV. DFT indicates the minimum amount of energy needed to return normal rhythm to a heart that is beating in a cardiac dysrhythmia. The SDVF‐DFT was determined with a three‐crossing bracketing protocol described in our previous study (Jin et al., 2013). Biphasic shocks (6/4 ms) were delivered from a defibrillator (Teletronic Pacing Systems, 4510 Implant Support Device, Teletronics Pty Limited, Malvern, Australia) via the defibrillating catheter. The leading‐edge voltage of the first shock was 400 V for the first animal. Depending on the success or failure of the shock, the leading‐edge voltage was decreased or increased by 40 V, respectively. The transition from failure to success or success to failure was recorded as the first data point. The up–down algorithm was continued until the third reversal of success to failure or failure to success was reached. The SDVF‐DFT was determined by averaging the four shock strengths that were required for the three reversals (Huang et al., 2002). At least 5 min elapsed after each SDVF episode to allow the BP and heart rate to return to normal.

After 20 s, the SDVF‐DFT was determined, and a 7 min LDVF episode was induced. LDVF‐DFT measurements were made as follows. The initial shock was given starting with 50% of the voltage of the SDVF‐DFT. A shock was considered successful if no VF was observed within 5 s after the shock. More than 5 s asystole was defined as persistent asystole. If VF was not terminated, repeat shocks with escalating voltage by 80 V increments were delivered until defibrillation success occurred. The lowest successful shock voltage was defined as the LDVF‐DFT.

### 2.5 Refibrillation

Refibrillation was defined as VF occurring more than 5 s after successful defibrillation. If return of spontaneous circulation (ROSC) was not observed within 10–20 s following defibrillation, direct cardiac compression was initiated. ROSC was defined as an organized electrical rhythm with a systolic BP of at least 60 mmHg for at least 1 min continuously at any time during the resuscitation effort. Compression was interrupted briefly after 1 min for reassessment of spontaneous circulation. In the episodes where refibrillation occurred, defibrillation was performed after 1 min of compressions. Basket electrode recordings were then made for 15 min to document any refibrillation episodes after the first successful defibrillation following the 7 min LDVF.

### 2.6 VF activation rate

The VF activation rate was estimated by fast Fourier transform (FFT) analysis of the VF at each electrode of the basket catheter in the RV and LV. The frequency with the highest power between 1 and 20 Hz was taken as the activation rate. The SDVF activation rate was calculated with an average of 20 s. The last 20 s of the 7 min LDVF was sampled to measure the LDVF activation rate.

### 2.7 Flunarizine administration

The control group was given normal saline (10 ml kg^−1^ h^−1^ maintenance). In the flunarizine group, after the baseline parameters were measured, flunarizine was administered intravenously in six animals (loading dose of 2 mg kg^−1^ over 10 min, maintenance dose of 4 mg kg^−1^ h^−1^). Thirty minutes after giving the loading dose, the parameters were again determined. The infusion of flunarizine was maintained until the end of the experiment.

### 2.8 Statistical analysis

Continuous variables are expressed as the mean ± SD, and categorical variables are expressed as a percentage. One‐way analysis of variance (ANOVA) with repeated measurements was used to compare the continuous variables from the two groups. Categorical data were analysed using the chi‐square test or Fisher's exact test where appropriate. For all analyses, *P *< 0.05 was considered statistically significant. All analyses were performed using the statistical software SAS version 9.2 (SAS Institute, Cary, NC, USA).

## 3 RESULTS

### 3.1 Effects of flunarizine on basic electrophysiology parameters

Table 1 summarizes the outcomes of flunarizine on the basic electrophysiological parameters. After administration of flunarizine, the systolic BP was significantly decreased by 22% (*P* < 0.001). The drug mildly reduced the sinus rate by 6% without statistical significance. Additionally, the QT and QTc intervals were not changed by flunarizine. Compared to baseline, the drug did not significantly alter the VERP during the S1S2 stimulation program. Consistent with the drug's effect on VERP, the mean ARI measured from the LV basket catheter was lengthened by only 5% after flunarizine (*P *> 0.05). Compared to the control group, the systolic BP after successful defibrillation following LDVF was significantly decreased in the flunarizine group (82 ± 10 *vs*. 71 ± 8 mmHg, *P* < 0.05).

**Table 1:** Basic electrophysiology parameters in the flunarizine group

	Before flunarizine	After flunarizine	
Parameter	(*n* = 6)	(*n* = 6)	*P*
Systolic BP (mmHg)	118 ± 12	92 ± 13	<0.001
Heart rate (beats min^−1^)	140 ± 14	131 ± 15	>0.05
QT interval (ms)	227 ± 22	234 ± 13	>0.05
QTc interval (ms)	346 ± 29	344 ± 28	>0.05
VERP (ms)	171 ± 5	180 ± 9	>0.05
ARI (ms)	150 ± 7	157 ± 12	>0.05

### 3.2 Altered VF activation rate by flunarizine

In the control group, the mean activation rate of SDVF in the RV was slower than that in the LV (*P* < 0.05) (Table 2). Compared to the control group, flunarizine did not significantly change the SDVF activation rate of either RV or LV. As VF continued, the activation rate of the 7 min LDVF was markedly reduced by 57% in the RV (*P* < 0.001) and by 59% in the LV (*P* < 0.001) in the control group. The reduced activation rate with the duration of VF in the flunarizine group was similar to that in the control group. However, compared to the control group, flunarizine significantly reduced the activation rate of LDVF by 23% in the RV (*P* < 0.01) and by 19% in the LV (*P* < 0.05).

**Table 2:** Altered VF activation rate during SDVF and LDVF by flunarizine

		Control group	Flunarizine group	
		(*n* = 6)	(*n* = 6)	*P*
SDVF activation rate (no. s^−1^)	RV	9.4 ± 1.0	9.2 ± 1.2	>0.05
	LV	10.3 ± 1.1	10.1 ± 1.0	>0.05
LDVF activation rate (no. s^−1^)	RV	4.0 ± 0.50	3.1 ± 0.46	<0.01
	LV	4.2 ± 0.59	3.4 ± 0.57	<0.05

### 3.3 DFT during different stages of VF

**Figure 1:**
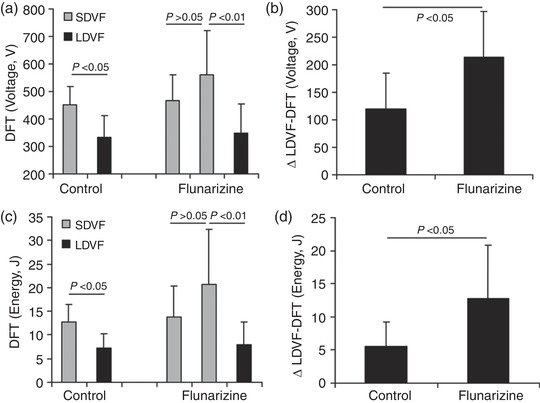
Defibrillation efficacy of flunarizine during different stages of VF. Mean DFTs and ΔLDVF‐DFT at different stages of VF in the control group (*n* = 6) and the flunarizine group (*n* = 6) (a,b, voltage; c,d, energy) are shown with the standard deviation indicated by an error bar. In (a,c), in the flunarizine group, the first and second grey columns indicate before and after flunarizine, respectively

In the control group, the mean DFT voltage and energy obtained after the 7 min LDVF (333 ± 79 V, 7.3 ± 3 J) were significantly lower than those obtained after SDVF (453 ± 65 V, *P *< 0.05; 12.8 ± 3.6 J, *P *< 0.05) (Figure 1a,c). In the flunarizine group, the voltage and energy of the SDVF‐DFT before and after the drug were not significantly different. Compared to the SDVF‐DFT after flunarizine, the voltage and energy of the LDVF‐DFT were significantly decreased by 38% (*P *< 0.01) and 36% (*P *< 0.01), respectively (Figure 1). The number of shocks delivered in the same ramp‐up protocol was 2.17 ± 0.75 and 2.0 ± 0.63 for the control group and flunarizine group, respectively (*P *> 0.05). However, the reduced value between the SDVF‐DFT and LDVF‐DFT (ΔLDVF‐DFT) after flunarizine was larger than that in the control group (213 ± 65 *vs*. 120 ± 84 V, *P *< 0.05; 12.7 ± 8.1 *vs*. 5.5 ± 3.7 J, *P *< 0.05) (Figure 1b,d).

### 3.4 Refibrillation following successful defibrillation of LDVF

**Figure 2:**
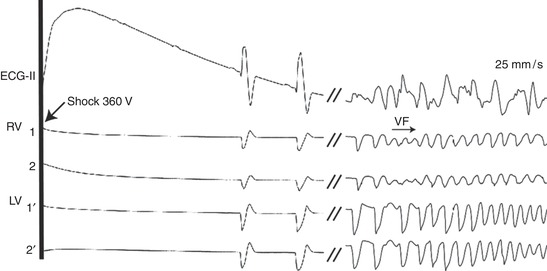
Refibrillation episode following successful defibrillation of LDVF. In one animal of the control group (data from animal no. 6), the shock (360 V in strength) initially terminated VF. Early refibrillation occurred after ventricular escape beats. RV1,2, two sites recorded by basket catheter in RV; LV 1′, 2′, two sites recorded by basket catheter in LV

There was no refibrillation episode after successful defibrillation of the 20 s SDVF in either the control group or the flunarizine group. However, early VF recurrence frequently occurred during LDVF in the control group. At least one refibrillation episode followed the 7 min LDVF in six animals. The refibrillation incidence of the 7 min LDVF after successful shock was significantly higher than that following the SDVF (0/18 *vs*. 6/6, *P *< 0.01) in the control group. In the flunarizine group, 5 out of 6 animals had more than one VF recurrence following LDVF after administration of flunarizine. The number of refibrillation episodes per animal (1.3 ± 1.0) following successful defibrillation of LDVF after flunarizine was 48% of that for controls (2.7 ± 2.0, *P *< 0.05). All of the first episodes of refibrillation occurred within the first 3 min in both groups. Figure 2 shows an early refibrillation after successful shock of 7 min LDVF in a representative animal of the control group.

**Figure 3:**
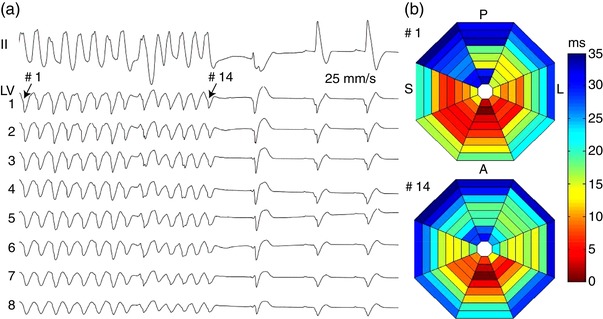
An example of ventricular tachycardia termination after successful defibrillation of LDVF. (a) VT in lead II of the ECG, one of the basket splines of LV after successful defibrillation of the 7 min LDVF in one animal (data from no. 3) in the flunarizine group. (b) Activation maps showing that the first and 14th beats of VT led by focal activity both originate from the apical‐anterior side. The colours represent activation times of the 64‐basket electrodes according to the time scale shown to the right. A, anterior free wall; L, left free wall; P, posterior free wall; S, septum. Apical electrodes are located towards the centre of the display, and basal electrodes are towards the periphery

In the control group, after six episodes of LDVF were initially halted by the shock, refibrillation occurred following ventricular escape beats. These beats then triggered focal activities and early VF recurrence. Interestingly, in four animals of the flunarizine group, after the first successful defibrillation of LDVF, four VT episodes occurred and were terminated but did not degenerate into VF during resuscitation (Figure 3). Figure 3a shows successful defibrillation of the 7 min LDVF in one animal in the flunarizine group. Figure 3b is a typical example showing that the VT after LDVF defibrillation was driven by focal activities originating from the apical‐anterior side of LV, and these focal activities were terminated by giving flunarizine, a DAD blocker. This figure partially supports our hypothesis that DADs potentially contribute to refibrillation in a canine model of LDVF, and thus flunarizine markedly reduced early VF recurrence of LDVF. However, no spontaneous termination of VT episodes occurred in the six control animals (0/6 *vs*. 4/6, *P* < 0.05).

## 4 DISCUSSION

The main findings of the current study are as follows. (1) Flunarizine, a Ca^2+^ channel blocker, significantly reduced the activation of LDVF. (2) Flunarizine did not significantly change the SDVF‐DFT. However, the reduced value between the SDVF‐DFT and LDVF‐DFT after flunarizine was larger than that in the control group. The different effects of flunarizine on the SDVF‐DFT and LDVF‐DFT indicated that the role of DADs in the defibrillation mechanism may differ as VF continues. (3) Flunarizine significantly reduced early VF recurrence following LDVF, suggesting that DADs potentially contribute to refibrillation in a canine model of prolonged VF.

### 4.1 Altered DFT at different stages of VF by flunarizine

Recent studies have shown that the maintenance and termination of LDVF differ from the maintenance and termination of SDVF (Dosdall et al., 2010; Li et al., 2008). Generally, SDVF is thought to be maintained by re‐entry (Gray et al., 1995; Panfilov & Hogeweg, 1995). We showed that verapamil significantly reduced the incidence of re‐entry during VF in pigs (Jin et al., 2014). As VF continues, activations may be led by a focus originating from Purkinje fibre activities, including early after‐depolarizations, DADs and abnormal automaticity. Thus, the role of prolonged global ischaemia produced by LDVF in defibrillation efficacy would be changed. Additionally, any treatment targeting different mechanisms between SDVF and LDVF would exhibit different effects on defibrillation. Our previous study has shown that the combination of sotalol and verapamil increased the DFT but accelerated LDVF termination (Jin et al., 2018). In the current study, flunarizine slightly increased SDVF‐DFT without statistical significance. However, ΔLDVF‐DFT after flunarizine was larger than that in the control group (12.7 ± 8.1 *vs*. 5.5 ± 3.7 J, *P *< 0.05) (Figure 1), indicating that the drug decreased the LDVF‐DFT more than the decrease observed without giving flunarizine. This finding suggests that DADs play a different role in the defibrillation mechanism and efficacy between SDVF and LDVF. For LDVF, DADs could be the origin of activations arising after failed near‐DFT shocks before degeneration back into VF. This result was consistent with the effect of flunarizine on the activation rate of SDVF and LDVF in this study (Table 2). Flunarizine slows ventricular arrhythmias resulting from DADs but accelerates rhythms based on automaticity (Vos et al., 1990). The drug significantly reduced the activation of LDVF without a change in the SDVF activation rate, indicating that DADs are more involved in LDVF than in SDVF. Thus, our study provides new evidence to support the theory that the maintenance and termination of LDVF differ from those of SDVF by giving flunarizine.

### 4.2 Effect of flunarizine on refibrillation following LDVF

TAs are generally thought to be a mechanism for the initiation of cardiac arrhythmias. A mechanism of TAs is spontaneous SR Ca^2+^ release, which causes Na^+^‐Ca^2+^ exchanger current activation and membrane depolarization, resulting in DADs (Bers, 2008). When DADs reach a threshold, they initiate TAs and arrhythmia (ter Boyden & ter Keurs, 2001; Keurs, Zhang, & Miura, 1998). Prevention of arrhythmogenic Ca^2+^ release can inhibit spontaneous VF and reduce VF inducibility in diseased rabbit hearts (Chou et al., 2014). Persistent calcium elevation during late phase 3 and phase 4 of a shortened action potential can lead to after‐depolarizations and spontaneous VF after successful defibrillation (Ogawa et al., 2009). Flunarizine, a Ca^2+^ channel blocker, has been documented to inhibit DAD‐induced TAs (Vos et al., 1990). In the current study, flunarizine decreased refibrillation episodes following successful defibrillation of LDVF by half in the controls (1.3 ± 1.0 *vs*. 2.7 ± 2.0, *P *< 0.05). Several factors can be related to this decrease by flunarizine. First, mapping data showed that the VF wave fronts during prolonged global ischaemia produced by LDVF were initially halted by shock, followed by one to five ventricular escape beats. These beats then triggered focal activities and early VF recurrence. Our study showed that spontaneous ventricular arrhythmias following successful defibrillation of LDVF were converted to non‐ventricular rhythms by the drug rather than degenerating to VF, as shown in the controls (Figure 3), which could potentially decrease VF recurrence (Wu et al., 2008). Second, the maintenance of LDVF changed as VF continued. Focal activities originating from Purkinje fibres played an important role in the maintenance of LDVF (Li et al., 2013; Li et al., 2008). The vulnerability to DADs was higher in Purkinje fibres than in other types of cardiac myocytes (Ferrier, 1977). Meanwhile, most of the focal activities (73%) before and after successful shock arose from the same location near the interventricular septum where Purkinje fibres were located (Wu et al., 2008). Therefore, after giving flunarizine, DADs in LDVF were inhibited before shock. Then, electrical shock in combination with the drug can suppress DAD‐induced focal activities arising from the same location before shock and thus decrease early VF recurrence.

### 4.3 Clinical implication

To date, electrical shock is the most effective strategy for terminating either SDVF or LDVF; however, electrical shock by itself can neither change the causes of nor eliminate the substrates of VF. Refibrillation, commonly observed even after successful defibrillation of prolonged VF, is associated with poor clinical outcomes of SCA during resuscitation (van Alem et al., 2003). In this study, flunarizine markedly reduced early VF recurrence of LDVF, suggesting that DAD‐induced TAs can be one of the substrates of refibrillation in healthy hearts. Therefore, DAD inhibition can be used as an adjunctive therapy for electrical defibrillation for treating prolonged VF. This finding provides evidence of efficacy for suppressing refibrillation that may have clinical implications for human defibrillation of LDVF with acute intravenous flunarizine. Certainly, administrating flunarizine cannot thoroughly eliminate the substrates of refibrillation of LDVF. Due to the complexity of refibrillation, it appears unlikely that DADs are the sole mechanism of refibrillation. Thus, electrical defibrillation combined with other drugs or interventional strategies such as pacing to target various substrates may decrease refibrillation episodes during resuscitation. These ideas require more experimental testing before translating into clinical resuscitation for patients with recurrent VF.

### 4.4 Limitations

The limitations of this study are as follows. First, although the RV and LV endocardium were simultaneously mapped, intramural recordings were not made. However, TAs were found frequently originating from endocardial surfaces but not from epicardial surfaces (Maruyama et al., 2010). Second, in this study, the shock voltage and energy from the constant voltage device were measured. The current level has been reported as the primary determination of defibrillation effectiveness. However, previous studies have shown that there were no significant impedance changes for shocks delivered from a fixed lead configuration on individual animals. Another limitation was that the current study was conducted in healthy hearts. Although a previous mapping study has demonstrated that LDVF by itself can cause refibrillation without requiring pre‐existing disease (Allred et al., 2008), the substrates of LDVF in pathological conditions such as myocardial infarction or heart failure could be altered. Thus, the role of DADs in defibrillation efficacy and refibrillation of LDVF in pathological heart models should be re‐evaluated. Third, a period of high‐quality cardiopulmonary resuscitation (CPR), by providing myocardial perfusion, would alter the VF waveform characteristics and consequently the likelihood of successful defibrillation. Therefore, this is one of the limitations of this study. Further studies should be designed to investigate the effect of flunarizine combined with standard CPR on LDVF defibrillation.

## 5 CONCLUSIONS

The effect of flunarizine on SDVF‐DFT and LDVF‐DFT indicates that the role of DADs in the defibrillation mechanism may differ as VF continues. Flunarizine significantly reduced early VF recurrence following LDVF, suggesting that DADs potentially contribute to refibrillation in a canine model of prolonged VF.

## COMPETING INTERESTS

The authors have no conflicts of interest to declare.

## AUTHOR CONTRIBUTIONS

C.F.X. and Q.J. designed the study, carried out the electrophysiological mapping study, and drafted the manuscript. N.Z. and S.H.L. carried out the mapping study, collected the data and performed the statistical analyses. C.J.L., Q.W., Q.Z.L. and A.L. carried out the mapping study. Q.J. and L.Q.W. conceived the study, participated in its design and coordination, and helped critically revise the manuscript for important intellectual content. All authors have read and approved the final version of this manuscript and agree to be accountable for all aspects of the work in ensuring that questions related to the accuracy or integrity of any part of the work are appropriately investigated and resolved. All persons designated as authors qualify for authorship, and all those who qualify for authorship are listed.
